# Application of the Reuterin System as Food Preservative or Health-Promoting Agent: A Critical Review

**DOI:** 10.3390/foods11244000

**Published:** 2022-12-10

**Authors:** Mao-Cheng Sun, Zi-Yi Hu, Dian-Dian Li, Yu-Xin Chen, Jing-Hui Xi, Chang-Hui Zhao

**Affiliations:** 1 College of Plant Science, Jilin University, Changchun 130062, China; 2 College of Food Science and Engineering, Changchun University, Changchun 130022, China; 3 Institute of Biological and Medical Engineering, Guangdong Academy of Sciences, Guangzhou 510316, China; 4 College of Food Science and Engineering, Jilin University, Changchun 130062, China

**Keywords:** reuterin, biosynthesis, antimicrobial mechanism, toxicity, preservative

## Abstract

The reuterin system is a complex multi-component antimicrobial system produced by *Limosilactobacillus reuteri* by metabolizing glycerol. The system mainly includes 3-hydroxypropionaldehyde (3-HPA, reuterin), 3-HPA dimer, 3-HPA hydrate, acrolein and 3-hydroxypropionic acid, and has great potential to be applied in the food and medical industries due to its functional versatility. It has been reported that the reuterin system possesses regulation of intestinal flora and anti-infection, anti-inflammatory and anti-cancer activities. Typically, the reuterin system exerts strong broad-spectrum antimicrobial properties. However, the antimicrobial mechanism of the reuterin system remains unclear, and its toxicity is still controversial. This paper presents an updated review on the biosynthesis, composition, biological production, antimicrobial mechanisms, stability, toxicity and potential applications of the reuterin system. Challenges and opportunities of the use of the reuterin system as a food preservative or health-promoting agent are also discussed. The present work will allow researchers to accelerate their studies toward solving critical challenges obstructing industrial applications of the reuterin system.

## 1. Introduction

In recent years, negative public perception of chemical preservatives prompts an increasing preference of consumers for “natural” agents from biological systems as alternatives [1,2]. To meet the consumers’ demand for “natural preservatives”, essential oils extracted from plants [3,4], bacteriocins from lactic acid bacteria such as nisin or pediocin PAß1-AcH and bacteriocin-producing protective cultures such as *Carnobacterium maltaromaticum* UAL307 (Micocin^®^ [5,6]) are used commercially as food preservatives. However, high production cost, narrow antimicrobial spectrum and limited antimicrobial activity challenge the application of these “natural preservatives” [2]. Further improvement in food safety and quality still necessitates the development of novel antimicrobial agents from natural origins.

The reuterin system is a multi-component antimicrobial complex produced by *Limosilactobacillus reuteri* by metabolizing glycerol. Reuterin was first discovered in the 1980s by Axelsso et al. [7], who observed that resting cells of *L. reuteri* converted glycerol into a low-molecular-weight, non-protein, antimicrobial substance, which was later referred as reuterin. Further research verified that reuterin is a dynamic multi-compound equilibrated system consisting mainly of 3-hydroxypropionaldehyde(3-HPA), its dimer, its hydrate and acrolein in aqueous solution [8,9]. Reuterin generally refers to 3-HPA, because 3-HPA is not only the precursor of other reuterin compounds but also considered to be the main antimicrobial component in the system. Among the reuterin-producing bacteria, including *Bacillus*, *Klebsiella*, *Citrobacter*, *Enterobacter*, *Clostridium* and *Lactobacillus* [10], the food-grade *L. reuteri* strains are the most suitable for reuterin production at industrial scale due to their high availability, probiotic properties, safety and high yield. Reuterin is generally produced by a two-step process using resting *L. reuteri* cultured with a glycerol–water solution, in which the yield is more than 3 times greater than that of the one-step process [11]. Quantification of 3-HPA can be achieved by colorimetric methods, HPLC, LC–MS, IC-PAD, etc., while lack of commercialized 3-HPA may affect the accuracy of measurement. The antimicrobial mechanism of the reuterin system remains unclear due to the complexity and dynamic nature of the system. Nevertheless, the reuterin system exhibits a broad-spectrum antimicrobial activity against pathogens and spoilage organisms, including Gram-positive and Gram-negative bacteria, yeasts, fungi, protozoa, viruses [12] and even antibiotic-resistant bacteria [13,14]. Gram-negative bacteria are usually more sensitive to the reuterin system than Gram-positive bacteria [15,16,17]. Remarkably, most *Lactobacillus* species are less sensitive to reuterin, especially *L. reuteri* [18]. Antimicrobial activities of the reuterin system *in vitro* have been well-reviewed by Stevens et al. [12]. For example, the minimal inhibitory concentration (MIC) of 3-HPA on genera of *Enterococcus*, *Eubacterium* and *Bacteroides* is as low as 7.5 mM.

The reuterin system exhibits antimicrobial activities in different food matrices including dairy products and meat [19,20]. Moreover, the system also has some potential health-promoting effects, such as conjugating heterocyclic amines, regulating the gut microbiota and anti-infection [21,22,23]. However, many factors still limit the application of the reuterin system at industrial scales. This paper aims to provide a critical review of the reuterin system including its biosynthesis, composition, biological production, antimicrobial mechanism, potential applications as a food preservative or health-promoting agent as well as challenges in industrial production of the reuterin system for commercial purposes, which may stimulate new ideas and suggestions toward its practical use.

## 2. Biosynthesis and Composition of the Reuterin System

**Figure 1:**
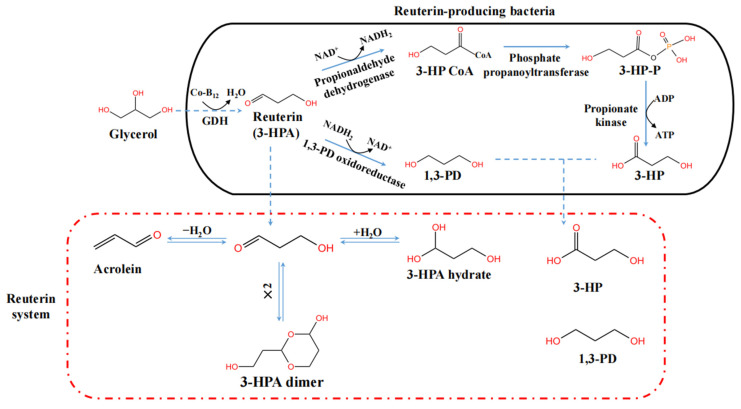
The metabolic pathway of reuterin (3-HPA) and the components of the reuterin system formed by *in situ* production of 3-HPA in the aqueous solution [12,26,31]. The reuterin system produced by 3-HPA purification does not contain 3-HP and 1,3-PD.

*L. reuteri* converts glycerol, as the electron acceptor, into an intermediate compound 3-HPA by the coenzyme B_12_-dependent glycerol dehydratase (GDH) in cells under anaerobic conditions. GDH exists as a dimer of subunits α2, β2 and γ2. The nucleotide and amino acid sequences of GDH are highly conserved in *L. reuteri* [24,25]. After being excreted outside the cell, 3-HPA forms a multi-compound dynamic reuterin system in aqueous solution, which includes 3-HPA, 3-HPA hydrate, 3-HPA dimer and acrolein. Moreover, 3-HPA in the cell is further reduced to 1,3-propanediol (1,3-PD) by the 1,3-PD oxidoreductase (an NADH-linked dehydrogenase) and spontaneously transformed into a structural isomer of lactic acid, 3-hydroxypropionic acid (3-HP), in three stages. First, 3-HPA is converted to 3-hydroxypropionyl-CoA (3-HP CoA) by the propionaldehyde dehydrogenase. Second, 3-HP CoA is phosphorylated to 3-hydroxypropionyl phosphate (3-HP-P) by the phosphate propanoyltransferase. Finally, dephosphorylation of 3-HP-P leads to the formation of 3HP catalyzed by the propionate kinase [26]. Notably, 1,3-PD and 3-HP can also be secreted [12]. A detailed metabolic pathway of reuterin and the components of the reuterin system are shown in Figure 1.

**Figure 2:**
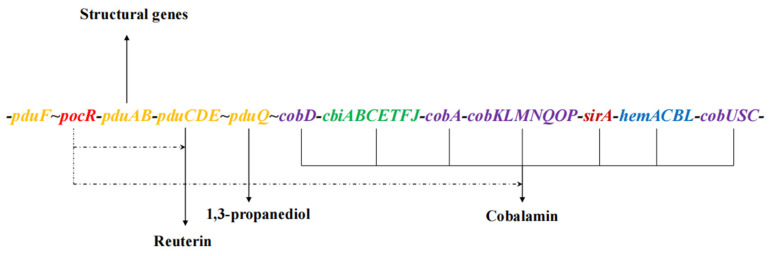
Genetic arrangement of reuterin and cobalamin in *L. reuteri* [27,28]. Yellow, *pdu* including *gupCDE* genes; Purple, *cob* genes; Green, *cbi* genes; Blue, *hem* genes; Red, *pocR*. The *pocR* gene regulates the synthesis of reuterin and cobalamin.

Gene islands are clusters of genes that are likely to be associated with the horizontal gene transfer events in bacteria. The genomic island in *L. reuteri* is believed to be related to the evolution of its probiotic properties in the human gut. The complicated biosynthesis of reuterin is determined by a genomic island containing a 1,2-propanediol utilization (*pdu*) cluster together with a vitamin B_12_ cluster (namely the *pdu*-*cbi*-*cob*-*hem* gene cluster) [27], which is proposed to be transcriptionally arranged as seen in Figure 2. The island harbors the *pocR* gene (lreu_1750)-encoding protein PocR that participates in both glycerol utilization and B_12_ biosynthesis [28]. The ability of *L. reuteri* to produce vitamin B_12_ is generally considered to be associated with 3-HPA production. Biosynthesis of 3-HPA from glycerol by vitamin B_12_-dependent glycerol dehydratase is encoded by *gupCDE* (*pduCDE*) in the *pdu* operon. [27]. The *pduF* gene encodes a hydrophobic protein that facilitates the diffusion of glycerol [29]. The *pduL*, *pduP* and *pduW* confer the transformation of 3-HPA to 3-HP [30]. The gene sets (*cbi*, *cob* and *hem*) that are involved in cobalamin biosynthesis are adjacent to the *pdu* operon [27].

## 3. Biotechnological Production of Reuterin

Reuterin (3-HPA) is the precursor of the reuterin system’s components. Although 3-HPA can be produced by chemical synthesis with acrolein dehydration, the reaction has to be completed at high temperature, high pressure and relatively high cost [10]. Biological production of 3-HPA has the advantage of mild reaction conditions, high yield and low cost compared with chemical synthesis [10]. Production of 3-HPA by fermentation is carried out by the *in situ* process or the two-step process. The former is usually achieved by metabolizing glycerol by *L. reuteri* in MRS medium, but low yield and a complicated purification process limit its application. The latter involves preparing a crude 3-HPA solution by a simple two-step process followed by purification [11]. Specifically, a large number of whole cells of *L. reuteri* are firstly collected from the MRS medium. Secondly, washed cells are incubated in a glycerol–water solution to produce 3-HPA. Finally, 3-HPA is purified by ion exchange resin, chromatography or reactive distillation methods [32,33,34,35].

There are some factors that affect the production of 3-HPA in the two-step process, such as glycerol concentration, biomass, incubation time, incubation temperature, etc. Bauer et al. [36] studied the effect of glycerol concentration (100~400 mM) on 3-HPA production and found that 300mM glycerol had the highest 3-HPA yield (27 mM) at a resting cell concentration of 1 × 10^9^ CFU/mL after 120 min of incubation at 20 °C, indicating that high concentrations of glycerol possibly inhibited the glycerol dehydratase activity of resting cells. *L. reuteri* was immobilized in spherical calcium alginate beads to increase 3-HPA productivity [37]. Moreover, the production of 3-HPA was expedited at pH 6, while no significant difference was found at incubation temperatures of 15 °C and 37 °C. Addition of lactate in the glycerol–water solution had a positive impact on the accumulation of 3-HPA while glucose showed the opposite result [38]. However, Doleyres et al. [11] reported that the maximum 3-HPA level (235 mM) was obtained using 400 mM glycerol, 1.6 × 10^10^ CFU/mL resting cells, incubation temperatures of 30 °C and an incubation time of 45 min in the glycerol–water solution. These parameters of optimal 3-HPA production are inconsistent and likely strain-dependent.

The challenge of 3-HPA production with glycerol is the toxicity of 3-HPA accumulated to *L. reuteri*. To solve this problem, bisulfite can be used to entrap 3-HPA to form a non-toxic 3-HPA–bisulfite complex that can markedly increase glycerol conversion by the resting cells. The produced 3-HPA can then be released from the complex by an ion exchange resin [39].

## 4. Antimicrobial Mechanisms

**Figure 3:**
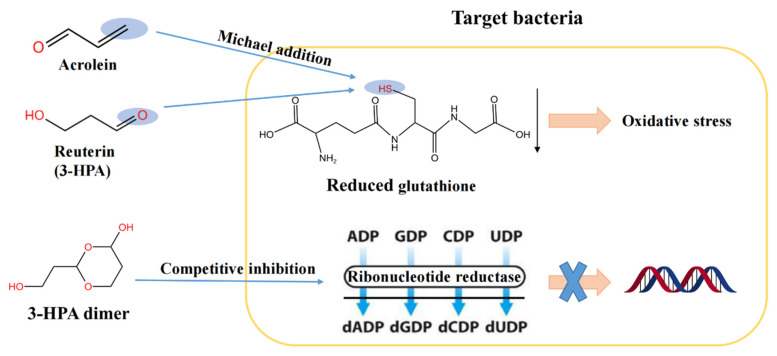
Three proposed antimicrobial mechanisms of the reuterin system [9,33,40,42].

Even though the antimicrobial mechanism of the reuterin system remains unclear and controversial, the structures of some antimicrobial compounds in the system have been analyzed and their antimicrobial mechanisms are initially proposed (Figure 3). The 3-HPA dimer may exhibit antimicrobial activity through acting as a competitive inhibitor of the ribonucleotide reductase that is responsible for DNA synthesis of cells [33]. The antimicrobial activity of 3-HPA may be attributed to the aldehyde groups of 3-HPA, which can induce oxidative damages to bacteria by depletion of free sulfhydryl groups in small proteins and other molecules such as reduced glutathione and enzymes [9,40]. Furthermore, 3-HPA at concentrations of more than 10 μM can downregulate the virulence gene expression in *Helicobacter pylori* including *vacA*, *flaA* and *luxS* [41]. Notably, acrolein may deplete the free sulfhydryl groups of reduced glutathione and other enzymes through a Michael addition reaction and, thus, may induce oxidative damages to bacteria and contribute to the antimicrobial activity of the reuterin system [42]. In addition, 3-HP acts synergistically with acrolein in inhibiting microbial growth [30,43]. Overall, the application of the reuterin system as a food preservative still necessitates more insights into its antimicrobial mechanisms.

## 5. Stability

The stability of the reuterin system is affected by temperature, media, pH, etc. Additionally, 3-HPA is highly soluble in water, resistant to proteases, lipases, nucleases and liable to heat (100 °C for 10 min) [12,44]. The 3-HPA content in the supernatant produced by the two-step process was stable at −20 °C for at least 35 days, and then it slowly decreased. Moreover, repeated freeze–thaw cycles (−20/4 °C) caused a slow decrease in 3-HPA with every cycle [23]. In addition, the degradation of 3-HPA to acrolein in aqueous solution was greater at high temperatures (37 °C and 40 °C), while there was very little degradation at 4 °C for 4 weeks [45]. Similarly, IC-PAD analysis suggested that 3-HPA/acrolein did not interconvert at low temperature and acidic pH [42].

3-HPA is much more stable in aqueous solution than that in milk or MRS media [46]. After 1 h of treatment at 100 °C, the level of 3-HPA in MRS produced by the one-step fermentation process significantly reduced, but was not notably affected in the glycerol–water solution produced by the two-step fermentation process [47]. After stirred yogurt was made from raw milk containing purified 3-HPA, 3-HPA level decreased by more than 90% during 15 days of storage at 21 °C, but the reduction was slower at 4 °C. This is likely due to the fact that 3-HPA can form Schiff bases with the proteins in milk [48]. Some examples concerning stability of the reuterin system are summarized in Table 1. 

## 6. Toxicity of the Reuterin System

The impact of the reuterin system on human health is critical to its application in the food and medical industries. In the cytotoxicity assay using the human hepatoma cell line HepG2, the purified 3-HPA was much less toxic than acrolein and only four times more toxic than the generally recognized as safe (GRAS) flavoring diacetyl, implying a low possibility of reuterin to cause *in vivo* disturbance [50]. In the assay using *in vitro* models of the gastrointestinal tract, the 3-HPA did not affect the viability or the membrane integrity of Caco-2 cells at less than 1080 mM. Furthermore, 3-HPA had no hemolytic effect on blood cells at 270 mM [51]. In addition, the purified 3-HPA at bactericidal concentrations of 40 mg/L did not exert any skin toxicity [52].

Acrolein (2-propenal) is the main toxicant in the reuterin system, and is easily converted from 3-HPA at moderate temperature. Acrolein is commonly found in foods, and also found in the human body from endogenous production and/or dietary routes [53]. The toxicity of acrolein alone has been well-studied. The World Health Organization has classified acrolein as a class 2A carcinogen, and established a provisional tolerable oral acrolein intake of 7.5 μg kg^−1^ bw day^−1^ for non-neoplastic effects [54]. Acrolein produced by GDH-competent intestinal bacteria by metabolizing glycerol can lead to the formation of the acrolein-DNA adduct, which may induce mutations in a model system containing resting cells of *L. reuteri* in combination with calf thymus DNA or bovine serum albumin. However, the concentration of glycerol needed to cause the acrolein-DNA adduct-mediated mutation was much higher than the physiological concentration [55]. Nevertheless, the association between the carcinogenicity of acrolein observed in cytotoxicity assays and human health remains hypothetical, and has not been verified in clinic trials yet [56]. Although the reuterin system appears to be safe based on the results from short-term toxicity tests, the dynamic nature and complexity of the system are worthy of more attention. Therefore, studies on the dynamic changes of the reuterin system and long-term toxicity evaluation are necessary for the safety use of reuterin.

## 7. Potential Applications of the Reuterin System

### 7.1. Food Preservative

The reuterin system has great potential as a food preservative due to its strong antimicrobial activity in various types of foods and high stability under different food-processing conditions. Reuterin can be used as a food preservative to extend the shelf life using the following approaches: (i) addition of 3-HPA-producing *Lactobacillus* and food-grade glycerol to the food to achieve *in situ* production of reuterin compounds, which does not require regulatory approval or label declarations [57], (ii) addition of purified, semi-purified or other forms of 3-HPA to the food, (iii) addition of 3-HPA to the food, followed by treatment with other antimicrobial hurdles.

#### 7.1.1. Dairy Products

The antimicrobial activity of the reuterin system has been validated in milk, yogurt and cheese (Table 2). Addition of *L. reuteri* and glycerol (50 mM) in milk, cheese and yogurt can produce sufficient 3-HPA to exert antimicrobial activity during manufacturing and storage [58]. Remarkably, addition of high concentrations of glycerol at more than 200 mM increased the redness of cheese, which may be attributed to the reaction of the excess glycerol with cheese components [59]. *In situ* production of 3-HPA prevented the late blowing defect caused by *Clostridium tyrobutyricum* in semi-hard ewe milk cheese [60,61]. Either *in situ*-produced or purified 3-HPA can exert antimicrobial effects in yogurt. Increased yellowish color was observed in the yogurt with 3-HPA at concentrations over 69 mM [17,62]. Nisin, lacticin 481 and enterocin AS-48 acted synergistically with 3-HPA in inactivating *Staphylococcus aureus* in milk, but this synergistic effect was not observed for *Escherichia coli* O157:H7, *Salmonella enterica*, *Yersinia enterocolitica*, *Aeromonas hydrophila* and *Campylobacter jejuni* [19]. These studies suggest that the reuterin system can be applied as a preservative in dairy products.

#### 7.1.2. Meat and Fish Products

Foodborne pathogens pose a serious threat to human health, and are easily transmitted to humans through contaminated meat products, especially raw or undercooked meat [71]. The use of the reuterin system can inactivate various foodborne pathogens in meat and meat products (Table 3). The reuterin system produced by *L. reuteri* completely killed *E. coli* O157:H7 by at least 3.0 log CFU/g in ground beef at 4 °C at day 20 [72]. The use of *L. reuteri* and glycerol as meat starter cultures reduced the cell counts of *E. coli* O157:H7 by at least 1.0 log CFU/g during fermentation [20]. *In situ*-produced 3-HPA also reduced *Campylobacter* isolated from chicken meat [31].

Purified 3-HPA inhibited the growth of *L. monocytogenes* on the surface of cooked pork and sausages at refrigerated conditions without any alteration in meat quality [78,79]. Single or combined use of purified 3-HPA and high hydrostatic pressure (HHP) at 450 MPa for 5 min inhibited the growth of *L. monocytogenes* in cold-smoked salmon [80,81]. These studies suggest that the reuterin system is able to improve the quality of meat and fish products.

#### 7.1.3. Other Products

Incorporation of 3-HPA in pectin-based edible coatings significantly reduced viable *Penicillium* spp. on strawberries by 2.0 log CFU/g without quality deterioration over 31 days of cold storage [82]. Washing fresh-cut lettuce with the reuterin solution containing 7.2–21.9 mM acrolein at 4 °C reduced *Enterobacteriaceae*, yeasts and molds by 2.1–2.8 log CFU/g and 1.3–2.0 log CFU/g, respectively. It needs to be noted that this treatment resulted in the discoloration of lettuce [83]. The undesirable discoloring effect of the reuterin system on vegetables is dose-dependent and limits the use of the reuterin system in fresh produce. The reuterin system is also not suitable as a preservative in bakery foods such as bread, because the baking process may eliminate its antimicrobial activity [47].

### 7.2. Health-Promoting Effects

Despite a lack of clinical trails, several *in vitro* and *in vivo* studies demonstrate that the reuterin system exhibits certain health-promoting effects including anti-infection, anti-inflammation and anti-cancer activities, presumably through interacting with host microbiota and through alterations in metabolome.

#### 7.2.1. Anti-Infective and Anti-Inflammatory Effect

Co-delivery of *L. reuteri* with glycerol was effective against *C. difficile* colonization in complex human fecal microbial communities, whereas the single use of glycerol or *L. reuteri* was ineffective. A global unbiased microbiome and metabolomics analysis confirmed that glycerol precursor delivery with *L. reuteri* altered the composition and function of the human microbial community that preferentially targeted *C. difficile* outgrowth and toxicity [84]. In a novel *in vitro* colonic fermentation model where adult immobilized fecal microbiota were treated with reuterin, the addition of glycerol alone or in combination with *L. reuteri* increased numbers of the *Lactobacillus*–*Enterococcus* group and decreased *E. coli* without affecting other bacterial populations. The selective decrease in *E. coli* populations may be due to *in situ* reuterin production. These findings indicate that the reuterin system can prevent intestinal infection [85]. Moreover, the effectiveness of 3-HPA produced by *L. reuteri* in protection against intestinal *Salmonella* infections was also validated using a three-dimensional organotypic model of human colonic epithelium [86].

The frequent emergence of antibiotic-resistant pathogens has become a major threat to public health worldwide. Presently, there is no report on the resistance of pathogenic microorganisms to the reuterin system. Reuterin was considered to have a bactericidal effect on methicillin-resistant *S. aureus* [13]. Bennett et al. [14] reported that reuterin was the most effective against multidrug-resistant bacteria causing bovine mastitis, compared with nisin, bactofein and pediocin. The reuterin system may be a new alternative to reduce the emergence of resistant organisms and treat infections caused by resistant organisms.

#### 7.2.2. Cancer Chemopreventive and Anticancer Effect

Heterocyclic aromatic amines (HAAs), such as 2-amino-1-methyl-6-phenylimidazo [4,5-*b*]pyridine (PhIP), are potentially highly mutagenic and/or carcinogenic food-derived contaminants formed in processed meat products. 3-HPA is produced by specific gut microorganisms from glycerol including *L. reuteri*, and then forms the reuterin system consisting of acrolein. Vanhaecke et al. [87] found that human intestinal bacteria were capable of transforming the dietary carcinogen PhIP into 7-hydroxy-5-methyl-3-phenyl-6,7,8,9-tetrahydropyrido [3′,2′:4,5]imidazo [1,2 a]pyrimidin-5-ium chloride (PhIP-M1) by the glycerol metabolite 3-HPA. Fekry et al. [88] suggested that the transformation of PhIP into PhIP-M1 is a detoxification process. Beer et al. [22] reported that acrolein in the reuterin system likely reacted with HAAs to form conjugates that blocked the exocyclic amino group of HAAs, thereby diminishing their ability to form DNA adducts that cause mutagenicity.

Recently, Bell et al. [89] reported that 3-HPA produced by *L. reuteri* in the healthy intestinal microbiome inhibited colon tumorigenesis, decreased tumor growth and prolonged survival in mice by inducing protein oxidation and inhibiting ribosomal biogenesis *in vivo* and *in vitro*.

#### 7.2.3. Fixation and Sanitization of Biological Tissues

3-HPA is more bactericidal but less cytotoxic than the most commonly used sterilant and crosslinker glutaraldehyde for biological tissues [90,91]. Although the rate of tissue fixation by 3-HPA is slower than that by glutaraldehyde, 3-HPA-fixed tissue has a reduced inflammatory reaction and comparable tensile strengths, calcification and resistance against degradation and free-amino group content [92,93]. Additionally, reuterin–polymerized pyridoxylated hemoglobin (RR-PLP-Hb) solution is a new option in the development of blood substitutes, because the solution has a lower oxygen affinity and longer vascular retention time than the unmodified Hb solution [94].

#### 7.2.4. Other Bioactivities

Ingestion of reuterin may also exhibit other health promoting effects. Oral administration of the reuterin supernatant altered the fecal microbiome, decreased heptane and increased 3-methylbutanal in the feces of mice, suggesting the high potential of the reuterin system to relieve some diseases caused by the dysbiosis of intestinal flora [23]. Oral treatment of broilers with water supplemented with a combination of 5 mM 3-HPA and 0.08 mM microcin J25 promoted growth performance and improved health, presumably through alterations in the cecal microbiome and metabolome, indicating the potential of 3-HPA as a growth promoter instead of as an antibiotic in animals [95]. Das et al. [96] found that 3-HPA effectively ameliorated iron overload diseases via suppressing hypoxia-inducible factor 2α (HIF-2α), a master transcription factor of intestinal iron absorption, using a high-throughput screen of gut microbial metabolites. More examples concerning health-promoting effects of the reuterin system are given in Table 4.

## 8. Conclusions and Future Perspectives

The reuterin system produced by *L. reuteri* is a multi-component dynamic equilibrium system that was discovered nearly forty years ago. The system has powerful broad-spectrum anti-microbial actions. Many factors can affect the composition of the system, such as temperature, pH and other chemical compositions. Due to the dynamic nature and lack of a fast, effective real-time assay, the contributions of specific substances in the system to the antimicrobial activity and modes of action have not been fully clarified. Thus, it is non-scientific to use only 3-HPA to represent the effective concentration of the system in most research. If the above problems are not solved, it will be difficult to achieve standardized production of commercial products of the reuterin system.

Numerous studies have shown the great potential of the reuterin system as a food preservative. However, most studies have only focused on the antibacterial effect and neglected its interaction with food components. For instance, there is a need to further investigate the reasons why the reuterin system tends to cause color changes in dairy products. Moreover, there are few research reports on the application of the system for vegetables, fruits and agriculture-based food products. Although the reuterin system has shown many health-promoting effects, *in vivo* and clinical studies are still lacking.

Conclusive toxicological data are the key to practical applications of the reuterin system in food and medicinal fields. The fate of the reuterin system from different sources should be fully evaluated, such as *in situ*-produced and purified 3-HPA. It is necessary to understand the metabolism and biotransformation pathways of the system in the body, including the skin, oral cavity, gastrointestinal tract, etc. Acute, subacute and long-term exposures of the system should be performed to obtain the safe dose range for various applications.

This review presents several insights into the dynamic nature of the reuterin system and provides critical examination on the use of the reuterin system as a food preservative or health promoting agent. Further research of the reuterin system should be carried out regarding production, antimicrobial mechanism, toxicity, bioactivities and its interactions with other food components. In addition, the efficacy of the reuterin system as a food preservative or health-boosting agent still needs to be validated in large-scale industries.

## Figures and Tables

**Table 1:** Stability of the reuterin system under different conditions.

Media	Source of the System	Reuterin Level	Detection Method	Main Observations	Refs
Glycerol aqueous solution	Supernatant produced by the 2-step method	13.4 g/L	HPLC	3-HPA remained stable for 35 days at −20 °C;	[23]
H_2_O, milk and MRS at various temperatures	Supernatant produced by the 2-step method	2.6–6.0 mM	Colorimetric assay, derivative spectroscopy	Compared with milk and MRS, 3-HPA in water was more stable in general.	[46]
Water at 37 °C at three pH values	Purified 3-HPA	Not stated	HPLC	3-HPA at pH 2 was more stable than at 6.5, while it degraded immediately at pH 11.	[49]
Cell culture condition	Purified 3-HPA	1.3 mM	HPLC	3-HPA remained stable after 24 h.	[50]
*In vitro* digestion model	Purified 3-HPA	Oral phase (270 mM/mL)	HPLC and colorimetric assay	No degradation or bioconversion of 3-HPA.	[51]

**Table 2:** The use of the reuterin system as preservative in dairy products.

Food	Target Bacteria	Source of the System	Reuterin Level	Combination with	Main Observations	Refs
Milk	*E. coli* O157:H7, *S.Enteritidis*, *L. monocytogenes*	Purified 3-HPA	1 AU/mL	Diacetyl (100 mg/kg)	The greatest inhibition of the 3 pathogens in acidified milk at pH 5.0.	[63]
Milk	*E. coli* O157:H7, *S. enterica*	Purified 3-HPA	8 AU/mL	Lactoperoxidase system (0.2 ABTSU/mL)	A strong synergistic bactericidal activity at 4 °C; complete elimination at 8 °C.	[64]
Milk	*Clostridium* strains	The supernatant produced by the 2-step method	0.06~32.50 mM	None	Growth inhibition of the vegetative cells and spores in MIC 0.51–32.5 mM after 7 d at 37 °C.	[65]
Stirred yoghurt	*Rhodotorula mucilaginosa,* *Aspergillus niger,* *Penicillium chrysogenum,* *Mucor racemosus*	Purified 3-HPA	10 mM	None	Significantly reduced their growth in yoghurt for 4 weeks at 4 °C.	[48]
Semi-hard cheese	*L. monocytogenes*, *E. coli O157:H7*	*In situ*	5.30 mM/g	None	The pathogen was not detected from day 7 onwards.	[66]
Semi-hard cheese	No antimicrobial activity assay	*In situ*	Not stated	None	Did not affect overall odor or aroma.	[67]
Semi-hard cheese	No antimicrobial activity assay	*In situ*	0.76~2.19 μM/g	None	Did not affect overall sensory scores for texture and color quality.	[68]
Semi-hard cheeses	*Clostridium tyrobutyricum*	*In situ*	Not stated	None	The vegetative cells were not detected, and did not cause late blowing defect.	[69]
Cottage Cheese	*L. monocytogenes*, *E. coli* O157:H7	Lyophilized 3-HPA produced by the 2-step method	50, 100, 150, 250 units/g	None	Reduced the viability of both organisms in 21 days at 7 °C.	[16]
Cuajada (curdled milk)	*Listeria monocytogenes*, *Staphylococcus aureus*	Purified 3-HPA	2 AU/mL	Nisin (100 IU/mL), Lactoperoxidase system (0.2 ABTSU/mL)	The pathogens were synergistically inactivated over 12 d at 10 °C.	[70]

**Table 3:** The use of the reuterin system as preservative in meat and fish products.

Food	Target Bacteria	Source of the System	Reuterin Level	Combination with	Main Observations	Refs
Sea bass fillets	*Pseudomonas* spp.	*In situ*	0.49, 0.55 g/L	None	The two active films containing reuterin showed a good antibacterial activity and improved microbiological and sensory quality of the fillets.	[73]
Chicken carcasses	*S. enterica*	The supernatant produced by the 2-step process	0.5, 2 mM	Lactic acid or microcin J25	Reuterin + lactic acid decreased *Salmonella* spp. counts by 2.02 log CFU/g and was more potent than reuterin + microcin J25.	[74]
Raw chicken legs	Total viable count (TVC)	The supernatant produced by the 2-step process	5 mM	Lactic acid or microcin J25	The combination delayed the growth of spoilage bacteria, extended the shelf life by 2–3 days, and maintained good sensory attributes at 4 °C for 10 days.	[75]
Cooked ham	*L. monocytgenes*, *S.* Enteritidis, *E. coli* O157:H7,	Purified 3-HPA	16 mM	HHP (450 MPa/5 min)	The combination inhibited the growth of the pathogens in the ham at 4 and 10 °C over 35 d.	[76]
Cooked ham	TVC	Purified 3-HPA	16 mM	HHP (450 MPa/5 min)	The combination reduced TVC and slightly affected color, texture and volatile compounds, but was worse than lactoperoxidase system + HHP over 35 days at 4 and 10 °C.	[77]

**Table 4:** The use of the reuterin system as health-promoting agent.

Bioactivities	Source of the System	Subject	Indices	Refs
Improving oral health	*In situ*	*In vitro*	Agar spot test against periodontal pathobionts and anaerobic commensals; oral biofilm formation; inflammatory expression of human oral keratinocytes.	[97]
Improving oral health	Reuterin analogues	*In vitro*	CH_3_SH production and detectable odor by *F. nucleatum* and *P. gingivalis*; the number of bacterial cells.	[98]
Regulation of intestinal microflora	*In situ*	*In vitro* (A chicken cecal model over 70 days)	Cecal microbiota composition and metabolism.	[99]
Treatment of gastrointestinal infections	*In situ*	*In vitro* and *in vivo* (cows, against O157:H7 EHEC)	Agar spot test and co-incubation; rumen fluid; rectal contents; feedstuff degradation.	[100]
Treatment of gastrointestinal infections	Purified 3-HPA and *in situ*	*In vitro* (against *C. perfringens*)	MIC; biofilm formation and motility; virulent gene expression.	[101]
Treatment of gastrointestinal infections	*In situ*	*In vitro* (a simulated gastrointestinal tract)	Antimicrobial assays using 96-well microtiter plates including *E. coli* O157:H7, *S. enterica*, *S. aureus* and *L. monocytogenes*.	[102]
Trypanocidal agent	The supernatant produced by the 2-step method	*In vitro*	The growth of culture form and motility, viability and macromolecular synthesis of culture and bloodstream forms of *Trypanosoma brucei.*	[103]
Trypanocidal agent	The supernatant produced by the 2-step method and purified 3-HPA	*In vivo* (7-week old female CF-I mice)	Toxicity; levels of parasitemia; survival of the mice.	[104]
Anti-necrotizing enterocolitis effect	The supernatant produced by the 2-step method	*In vivo* (SD rat pups)	NEC incidence; histology.	[105]
Anti-pullorum disease effect	*In situ*	*In vivo* (broiler chicks)	Anti-*Salmonella* chick experiment.	[21]
Treating colorectal cancers	Not stated	*In vitro* (colorectal cancer cells including HCT116, DLD1 and SW480)	MTT assay; colony forming assay; cell death assay; ROS assay.	[106]
Immunomodulatory activity	Purified 3-HPA	*In vitro* (LPS-induced chicken macrophage HD11 cells)	Cellular ROS; nitric oxide; oxidative stress indices; proinflammatory cytokines; signaling pathway.	[107]

## Data Availability

Not applicable.
